# APOBEC Mutagenesis Is Concordant between Tumor and Viral Genomes in HPV-Positive Head and Neck Squamous Cell Carcinoma

**DOI:** 10.3390/v13081666

**Published:** 2021-08-23

**Authors:** Daniel L. Faden, Krystle A. Lang Kuhs, Maoxuan Lin, Adam Langenbucher, Maisa Pinheiro, Meredith Yeager, Michael Cullen, Joseph F. Boland, Mia Steinberg, Sara Bass, James S. Lewis, Michael S. Lawrence, Robert L. Ferris, Lisa Mirabello

**Affiliations:** 1 Department of Otolaryngology-Head and Neck Surgery, Massachusetts Eye and Ear, Boston, MA 02118, USA; maoxuan_lin@broadinstitute.org; 2 Massachusetts General Hospital, Boston, MA 02118, USA; alangenbucher@mgh.harvard.edu (A.L.); mslawrence@mgh.harvard.edu (M.S.L.); 3 Harvard Medical School, Boston, MA 02115, USA; 4 Broad Institute of MIT and Harvard, Cambridge, MA 02142, USA; 5 Department of Epidemiology, University of Kentucky, Lexington, KY 40502, USA; Krystle.Kuhs@uky.edu; 6 Division of Cancer Epidemiology and Genetics, National Cancer Institute, National Institutes of Health, Rockville, MD 20847, USA; maisa.pinheiro@nih.gov (M.P.); yeagerm@mail.nih.gov (M.Y.); michael.cullen@nih.gov (M.C.); bolandj2@mail.nih.gov (J.F.B.); mia.steinberg@nih.gov (M.S.); sara.bass2@nih.gov (S.B.); mirabellol@mail.nih.gov (L.M.); 7 Cancer Genomics Research Laboratory, Leidos Biomedical Research, Inc., Frederick, MD 21701, USA; 8 Department of Pathology, Microbiology, and Immunology Vanderbilt University Medical Center, Nashville, TN 37011, USA; james.lewis@vumc.org; 9 Department of Otolaryngology, University of Pittsburgh, Pittsburgh, PA 15106, USA; ferrrl@upmc.edu; 10 Department of Immunology, University of Pittsburgh, Pittsburgh, PA 15106, USA; 11 UPMC Hillman Cancer Center, Pittsburgh, PA 15106, USA

**Keywords:** APOBEC, HPV, Humanpapilloma virus, oropharyngeal cancer, oropharyngeal squamous cell carcinoma

## Abstract

APOBEC is a mutagenic source in human papillomavirus (HPV)-mediated malignancies, including HPV+ oropharyngeal squamous cell carcinoma (HPV + OPSCC), and in HPV genomes. It is unknown why APOBEC mutations predominate in HPV + OPSCC, or if the APOBEC-induced mutations observed in both human cancers and HPV genomes are directly linked. We performed sequencing of host somatic exomes, transcriptomes, and HPV16 genomes from 79 HPV + OPSCC samples, quantifying APOBEC mutational burden and activity in both host and virus. APOBEC was the dominant mutational signature in somatic exomes. In viral genomes, there was a mean of five (range 0–29) mutations per genome. The mean of APOBEC mutations in viral genomes was one (range 0–5). Viral APOBEC mutations, compared to non-APOBEC mutations, were more likely to be low-variant allele fraction mutations, suggesting that APOBEC mutagenesis actively occurrs in viral genomes during infection. HPV16 APOBEC-induced mutation patterns in OPSCC were similar to those previously observed in cervical samples. Paired host and viral analyses revealed that APOBEC-enriched tumor samples had higher viral APOBEC mutation rates (*p* = 0.028), and APOBEC-associated RNA editing (*p* = 0.008), supporting the concept that APOBEC mutagenesis in host and viral genomes is directly linked and occurrs during infection. Using paired sequencing of host somatic exomes, transcriptomes, and viral genomes, we demonstrated for the first-time definitive evidence of concordance between tumor and viral APOBEC mutagenesis. This finding provides a missing link connecting APOBEC mutagenesis in host and virus and supports a common mechanism driving APOBEC dysregulation.

## 1. Introduction

The apolipoprotein-B mRNA-editing catalytic polypeptide-like (APOBEC) 3 family of cytidine deaminases is a major mutagenic source in human papillomavirus (HPV)-mediated cancers, including cervical and oropharyngeal squamous cell carcinoma (HPV + OPSCC) [1,2,3,4,5]. APOBEC-induced mutations constitute a high proportion of the somatic mutations in HPV + OPSCCs and can result in driver mutations, such as activating mutations in PIK3CA [3,6]. The underlying mechanisms driving APOBEC dysregulation and mutagenesis in HPV + OPSCC, and other HPV-mediated cancers, are unknown. Since APOBECs are viral restriction agents, it has been proposed that host genome mutations may actually be “collateral damage” following APOBEC activation by viral infecton [7,8].

Following HPV infection, APOBEC is upregulated by HPV oncoproteins and by IFN as part of the innate immune response, and this can inhibit HPV both directly, with the cytidine deamination of viral DNA leading to degradation, and indirectly, by decreasing HPV virion infectivity [9,10,11,12]. APOBEC-mediated mutational signatures have been identified in both viral and cancer genomes. Viral editing is identifiable and reproducible in vitro through APOBEC induction and in vivo in cervical pre-cancers and cancers [13,14,15,16]. APOBEC mutations are present in nearly all human cancers but are particularly prominent in HPV + OPSCC, where they are associated with multiple measures of immune upregulation [1,2,4,5,17,18,19,20].

We and others have shown that HPV lineages/sublineages and individual genetic variants are associated with important differences in cervical carcinogenicity [21,22,23,24,25,26,27,28,29,30,31,32,33,34,35]. In particular, our group has published large case–control studies characterizing mutations across cervical HPV16 genomes, utilizing an HPV whole genome sequencing (WGS) approach. These studies demonstrated that benign or clearing HPV16 infections have more genetic variants in specific viral regions than cervical precancers/cancers [34,36]. APOBEC-associated mutational signatures were also identified across the HPV16 genome and associated with a significant reduction in the carcinogenicity of HPV16 [36]. Thus, evidence supports the presence of APOBEC-induced mutations in both the genomes of HPV-mediated cancers and in HPV itself; however, we do not know whether APOBEC mutational activity in host and virus is directly linked. Here, using 79 HPV + OPSCC samples with paired sequencing of host somatic exomes, transcriptomes, and viral whole genomes, we apply computational approaches to characterize APOBEC activity in both host and viral genomes.

## 2. Materials and Methods

### 2.1. Samples

A total of 79 HPV + OPSCC tissue and paired blood samples were identified through existing databases at Vanderbilt University Medical Center and the University of Pittsburgh. HPV status was determined by p16 staining and subsequently by WGS for the detection of viral genomes. After the pathology review, DNA and RNA were extracted from FFPE tissue blocks using Zymo Quick DNA and RNA FFPE kits. Of these, 18 samples had been previously contributed to the Cancer Genome Atlas (TCGA) from the same institutions and had pre-existing whole exome sequencing (WES) and RNA-Seq. For these cases, DNA was extracted from FFPE blocks for viral WGS alone.

### 2.2. Sequencing

Somatic DNA underwent WES using the KAPA HyperPrep Library Preparation Kit, followed by hybrid capture with the Illumina Rapid Capture Exome Enrichment Kit. All sample pairs were validated with Fluidigm fingerprint data to confirm sample identity and fidelity. The average (range) coverage was 218× (70–402×). Somatic RNA underwent RNA-Seq using the Illumina TruSeq RNA Exome Kit. The average (range) number of reads per samples was 151 M (17.5 M–333 M). HPV16 DNA underwent WGS as previously described by custom AmpliSeq^TM^ panels, followed by sequencing on an Ion Torrent platform with average (range) coverage of 23,000× (1500×–56,000×) [37]. A total of 74 WES-seq, 68 RNA-seq, and 72 viral WGS samples passed quality control. Of these samples, 63 had data available from all three platforms.

### 2.3. Informatics

Somatic datasets: Somatic SNVs were called using MuTect v1. Mutations were screened against Panel of Normal (PoN) databases to remove common SNPs and recurrent sequencing artifacts. SNP-counting and copy number segmentation were carried out using FACETS. SNP counts were generated with a minimum mapping quality of 15, minimum base quality of 20, pseudo-spacing of 100 and a minimum read count of 25. Copy number data were segmented using a window size of 1000, Variant Allele Fraction (VAF) threshold of 0.3 and cval of 300. Allelic amplifications/deletions were defined as regions of any size with rounded integer total copy number states above or below 2, respectively. Somatic mutation lists were combined with mutations lists from the entire TCGA cohort, and mutational signatures were deconvolved using NMF (K = 8), as described previously [1,38]. The IFN-γ score was defined as the mean expression of six genes (IFNG, IDO1, CXCL9, CXCL10, HLA-DRA and STAT1) as described previously [39]. The analysis of the PI3K/AKT/mTOR pathway was conducted using a six major effector gene signature (PI3K, AKT1, AKT2, AKT3, PTEN, and MTOR) as described previously [40], and further, hotspot loci were examined in IGV. Significantly mutated genes were identified using MutSig2CV, and significantly copy-number-altered genes were identified using GISTIC 2.0. MutSig and GISTIC *p* values were combined using Fisher’s method to calculate a unified *p* value per gene. Pathway enrichment scores were then calculated by combining unified *p* values for genes in a pathway, also using Fisher’s method.

Viral datasets: All HPV16 WGS data were aligned to a consensus HPV16 sequence NC_001526.4 from PaVE [41]. Position-wise base counts were obtained, and a major haplotype was created by taking the most prevalent nucleotide at each position. These haplotypes were fed into the multi-alignment tool MUSCLE, along with multiple reference builds for HPV16 (AF402678, AF472509, AF534061, AF536179, AF536180, AY686579, HQ644236, HQ644257, HQ644298, K02718), and reference sequences for HPV35, HPV33, and HPV18. The resulting alignment was fed into PhyML (run using TOPALi v2.5), assuming a transversion model and allowing for invariable sites and specifying a gamma distribution. The resulting phylogenetic tree allowed for the sublineage assignment of each sample by nearest distance to a reference sequence. All samples were realigned with respect to their relative reference. Putative mutation sites were nominated by having a minimum total coverage of 100, a minimum alternate allele fraction of 0.02, and minimum alternate reads of 2. Putative mutations were anonymized and manually reviewed in IGV alongside other samples aligned to the same reference to remove low-evidence mutations and recurrent sequencing artifacts.

Viral APOBEC-induced mutations were identified as C->T and C->G mutations at TCW motifs (W is A or T), as described previously [36]. To count the number of APOBEC targetable sites, we first counted TCW motifs across the HPV16 genome for each reference build and then the total number across all samples’ HPV16 genomes on their respective reference builds. Since there are three possible changes at each nucleotide position, APOBEC-targetable sites were counted as one-third of the total number of TCW motifs. Mutation rates were calculated as the total number of APOBEC mutations, divided by the total number of APOBEC-targetable sites at the gene level and genome level, stratified by mutation type (synonymous (S) and non-synonymous (NS)) and variant allele fraction (VAF) (low-VAF (VAF ≤ 0.5) and high-VAF (VAF > 0.5)).

## 3. Results

**Figure 1:**
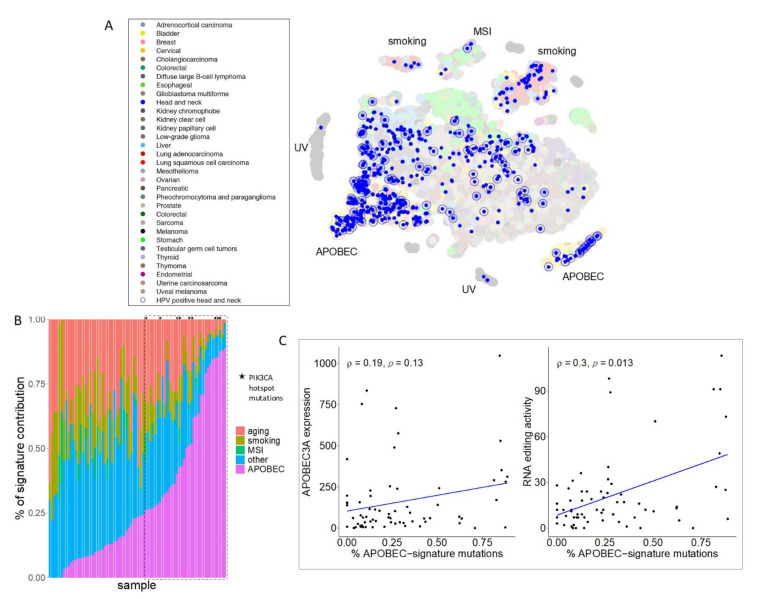
APOBEC is the dominant mutational signature in HPV + OPSCC. (**A**) Somatic mutation lists were combined with all cancers in TCGA and mutational signatures were deciphered using NMF. Mutational patterns were then projected into two-dimensional space with t-SNE. Head and neck squamous cell carcinoma tumors were colored in blue dots and HPV + OPSCCs were highlighted with blue circles. (**B**) Mutational signatures ordered by APOBEC contribution (magenta) in HPV+ OPSCC samples. PIK3CA hotspot mutations, E542K and E545K, were exclusively detected within APOBEC-enriched (>25%; dashed line box) tumors. (**C**) Weak correlations between APOBEC mutation burden and APOBEC3A (A3A) expression (**left**) and A3A-associated RNA editing activity (**right**).

A total of 79 HPV + OPSCC primary tumor samples were included in the analysis. The average patient age was 55. A total of 82% of the cohort were male and 60% had a history of tobacco exposure of >10 pack years. Somatic exomes were pooled with TCGA data and mutational signatures extracted. As expected, HPV + OPSCC samples predominately clustered with other tumors dominated by the APOBEC mutational signature, with variable APOBEC mutational burden between samples (Figure 1A,B, Supplemental Figure S1). PIK3CA hotspot mutations, which have previously been described to be caused by APOBEC activity in HPV + OPSCC, were exclusively associated with APOBEC enriched samples (Figure 1B). The PI3K/AKT/mTOR pathway was disproportionately mutated across the cohort (*p* < 3.22 × 10^−8^) and amplifications in chromosomes arm 3q, which contains PIK3CA, were present in 41 samples. Both of these alterations have previously been described to occur frequently in HPV + OPSCC^5^. APOBEC mutations, A3A expression, and APOBEC RNA editing, as measured by hotspot DDOST558C > U mutations, were weakly correlated, consistent with prior reports, likely due to the episodic nature of APOBEC activity (Figure 1C) [42].

**Figure 2:**
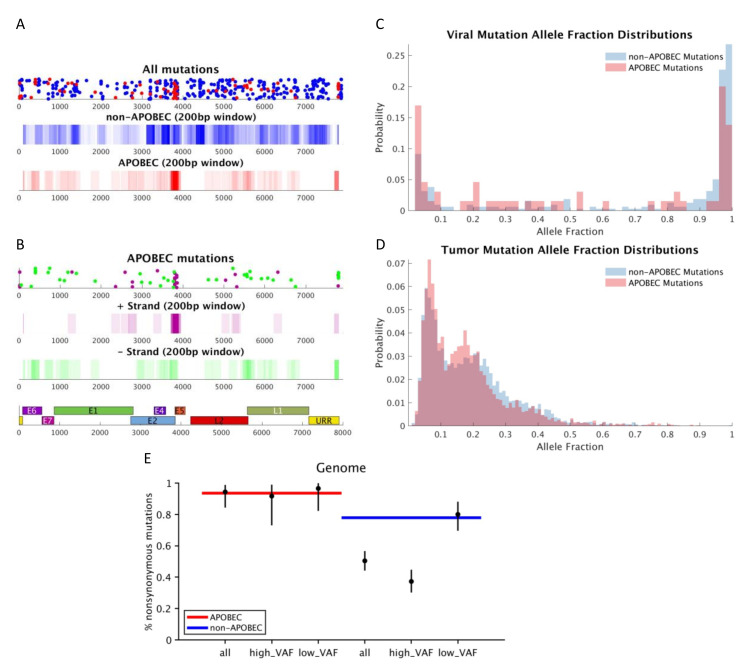
Mutation distributions in the viral and tumor genomes. (**A**) All viral mutations distributed across the genome with APOBEC mutations colored in red and non-APOBEC mutations in blue. (**B**) Distribution of viral APOBEC mutations, with positive strand mutations in purple and negative strands in green, highlighting enrichment of variants in E2. Viral (**C**) and tumor (**D**) APOBEC and non-APOBEC mutations by variant allele fraction demonstrating predominance of very low and very high VAF mutations in the viral genome. (**E**) Expected (blue and red lines) vs. actual (black dots) nonsynonymous/synonymous ratio for APOBEC (red) and non-APOBEC (blue) mutations demonstrating higher non-synonymous/synonymous ratios for APOBEC mutations and depletion of non-synonymous variants at high VAFs compared to expected in non-APOBEC mutations, suggesting purifying selection.

APOBEC mutations were annotated in the HPV16 genomes after first assigning each viral genome to a sublineage. A1 (*n* = 45) was the predominant sublineage, followed by A2 (*n* = 19), D3 (*n* = 4), A4 (*n* = 3) and C1 (*n* = 1). There was an average of five (range 0–29) mutations per viral genome. The mean APOBEC mutations in the viral genomes was one (range 0–5), with an APOBEC to non-APOBEC mutation ratio mean of 1:5. Variants were most heavily clustered in the E2 C-terminal DNA-binding domain (Q349E, S364C, S348C, E344Q), including in a position previously described [43] to impact transcription and the replication of the viral genome (D338N) (Figure 2A,B and Supplemental Table S1). There was no clear strand bias for HPV16 APOBEC mutations, suggesting that cytosines on both strands of the viral DNA are vulnerable to attack by APOBEC, likely during viral DNA replication, when both strands of viral DNA experience a single-stranded state. This is similar to APOBEC mutations in human DNA, which are also observed on both strands, but with a detectable bias toward the lagging-strand template, not observed here, possibly due to a lack of power owing to low overall viral mutation counts [44]. APOBEC mutations were found predominantly at very low and very high VAFs (Figure 2C,D). Viral APOBEC mutations, compared to non-APOBEC mutations, were more likely to be low-VAF mutations (occurring newly within the host), while non-APOBEC mutations were more likely to be high-VAF (existing prior to infection of the current host), suggesting that APOBEC mutagenesis actively occurrs in viral genomes during infection of the current host. Viral APOBEC mutations had a higher nonsynonymous/synonymous ratio, while non-APOBEC mutations showed the depletion of non-synonymous variants at high VAFs compared to expectation, suggesting the influence of purifying selection (Figure 2E, Supplemental Figure S2).

**Figure 3:**
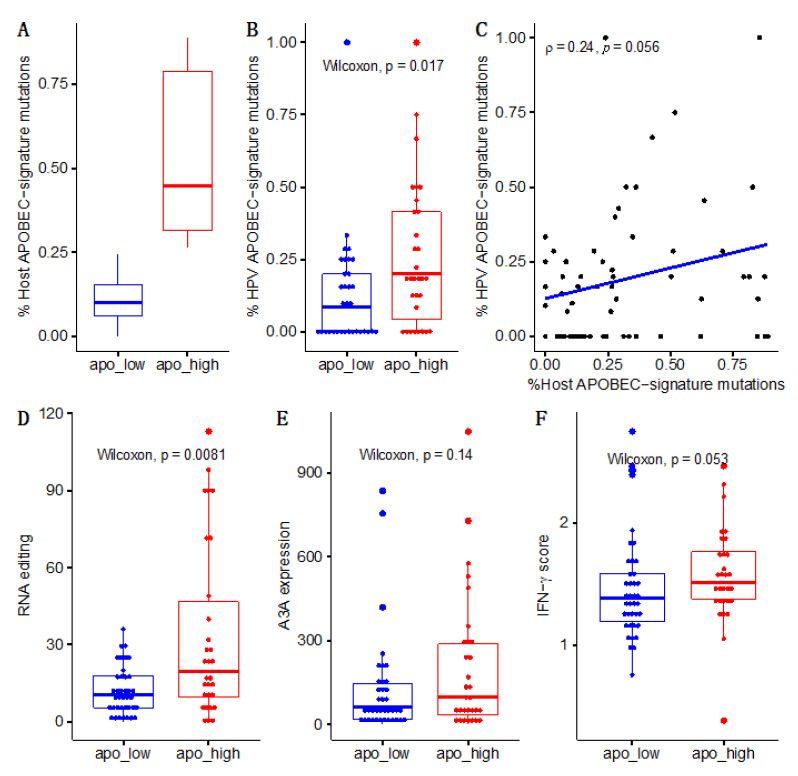
APOBEC mutations are concordant between tumor and viral genomes. APOBEC (**A**) mutations in tumor genomes, (**B**) mutations in the viral genome. (**C**) correlation between host and viral genomes, (**D**) RNA editing and (**E**) 3A gene expression, demonstrating linkage between APOBEC activity in virus and host. (**F**) APOBEC-enriched subjects also had higher IFN-γ scores, suggesting immune upregulation, possibly in response to viral infection.

To assess the relationship between APOBEC mutations in the viral and host genomes, samples were divided into APOBEC-low and -high mutational burden groupings based on host somatic mutations (Figure 3A). APOBEC-high samples had higher viral APOBEC mutation rates (*p* = 0.017; Figure 3B), and APOBEC mutagenesis in host and viral genomes was positively correlated (ρ = 0.24, *p* = 0.056; Figure 3C). We further examined additional measures of APOBEC activity in both tumor and virus and found similar trends for APOBEC RNA editing at the DDOST hairpin hotspot (*p* = 0.008), A3A mRNA expression (*p* = 0.14), and IFN-γ scores (*p* = 0.053), which are tied to APOBEC activity (Figure 3D–F).

## 4. Discussion

We conducted the first study to-date evaluating APOBEC-induced mutations in both host and viral genomes, providing a missing link connecting APOBEC mutagenesis in host and virus and supporting a common mechanism driving APOBEC dysregulation. Our group previously characterized HPV16 APOBEC-induced mutations in over 5000 cervical benign, pre-cancer and cancer samples, and identified low VAF mutations linked to benign or clearing infections [36]. We [36] and others [16] have suggested that the low VAF viral mutations were likely induced during infection. Here, we characterized HPV16 APOBEC-induced mutations in HPV + OPSCCs using deep NGS and utilized the paired sequencing of HPV16 and host somatic genomes to determine whether APOBEC activity was concordant in host and viral genomes. We showed that OPSCC HPV16 APOBEC-induced mutations occurred predominantly at low VAF, similar to what we observed in cervical HPV16 genomes, and importantly, correlated them with host somatic mutations. These data show that APOBEC mutates both cancer and viral genomes during an infection.

We identified APOBEC-signature mutations in OPSCC HPV16 genomes, which existed at both very low (new within host mutations) and very high (old mutations carried from prior host infections) VAFs. The OPSCC HPV16 APOBEC mutation patterns observed were similar to those previously reported in cervical HPV16 genomes [36]. Viral APOBEC mutations were most prominent at low VAFs, which is of interest as HPV genomes have long been considered stable during persistent infection, yet these data suggest that APOBEC editing of viral genomes is actively occurs during infection. This is despite the fact that HPVs have evolved to have fewer APOBEC target sequence sites, likely in an effort to avoid APOBEC restriction and thus viral damage and clearance [45]. This evolutionary pattern also results in the enrichment of potential non-synonymous APOBEC mutations compared to non-APOBEC mutations. In accordance with this, we observed a high nonsynonymous-to-synonymous ratio for viral APOBEC mutations overall. The densest cluster of APOBEC mutations occurred in E2 and included D338N, an HPV16 SNP that was previously reported to reduce p53 binding [46]. Interestingly, D338N was also prevalent in cervical cancer samples from our previous study. We also found a depletion of nonsynonymous high-VAF mutations for non-APOBEC mutations, but did not observe this for APOBEC mutations. This could suggest that some nonsynonymous viral APOBEC-induced mutations may be beneficial to the virus and contribute to the evasion of host immunity by altering viral antigens [7]. It is also possible that deleterious APOBEC mutations that result in viral damage and clearance are simply not detected, as those viruses have already been cleared during initial infection stages, leaving only viral genomes with a selective growth advantage present in the cancer.

Importantly, we found that APOBEC-enriched somatic samples (greatest APOBEC mutational burden and highest level of RNA editing) had higher viral APOBEC mutation rates, providing direct evidence that APOBEC mutagenesis in host and viral genomes is linked and occurs during infection. This suggests that in HPV-mediated cancers, the underlying processes driving APOBEC dysregulation and resultant TCW context mutations may be the same for both viral and somatic mutations. Mechanisms that drive APOBEC mutagenesis in HPV + OPSCC, however, remain unknown. While APOBEC is known to be activated by HPV oncoproteins, and through IFN signaling as part of innate immunity/viral nucleic acid sensing, APOBEC mutations are prevalent in non-virally mediated tumors, such as bladder, breast, and lung cancer, as well as non-HPV mediated head and neck cancers, albeit to a lesser degree [1,2,20]. Thus, it is possible that both viral/innate immunity and immune-independent processes, such as ssDNA targeting of the lagging strand during replication stress or after double strand breaks, drive APOBEC expression in HPV + OPSCC, and the high rates of APOBEC mutations in HPV + OPSCCs are a resultant cumulative effect. This concept is supported by work from our group and others showing multiple sources of APOBEC upregulation likely contribute to APOBEC mutagenesis in HPV + OPSCC, including APOBEC germline polymorphisms and immune upregulation in response to mutation-induced neoantigens [6].

In summary, we report the first study evaluating APOBEC mutagenesis in paired human somatic and viral genomes, identifying the presence of APOBEC mutations in OPSCC HPV genomes and concordance between APOBEC mutational burden within virus and host during infection. These data provide a missing link connecting APOBEC mutagenesis in host and virus and support a common mechanism driving APOBEC dysregulation.

## Data Availability

The datasets used and/or analyzed during the current study are available from the corresponding author on reasonable request.

## Supplementary Materials

The following are available online at https://www.mdpi.com/article/10.3390/v13081666/s1, Figure S1: Mutational signatures ordered by APOBEC contribution, Figure S2: Expected vs. actual nonsynonymous/synonymous ratio for APOBEC and non-APOBEC mutations by viral gene. Table S1: HPV E2 APOBEC Mutations.
