# Supplementary material for: APOBEC Mutagenesis Is Concordant between Tumor and Viral Genomes in HPV-Positive Head and Neck Squamous Cell Carcinoma
Source: Viruses. 2021 Aug 23;13(8):1666. doi: 10.3390/v13081666 (PMC8402723; doi:10.3390/v13081666)

Figure S1 Mutational signatures ordered by APOBEC contribution (magenta) in HPV+ OPSCC samples. Y axis= number of mutations.

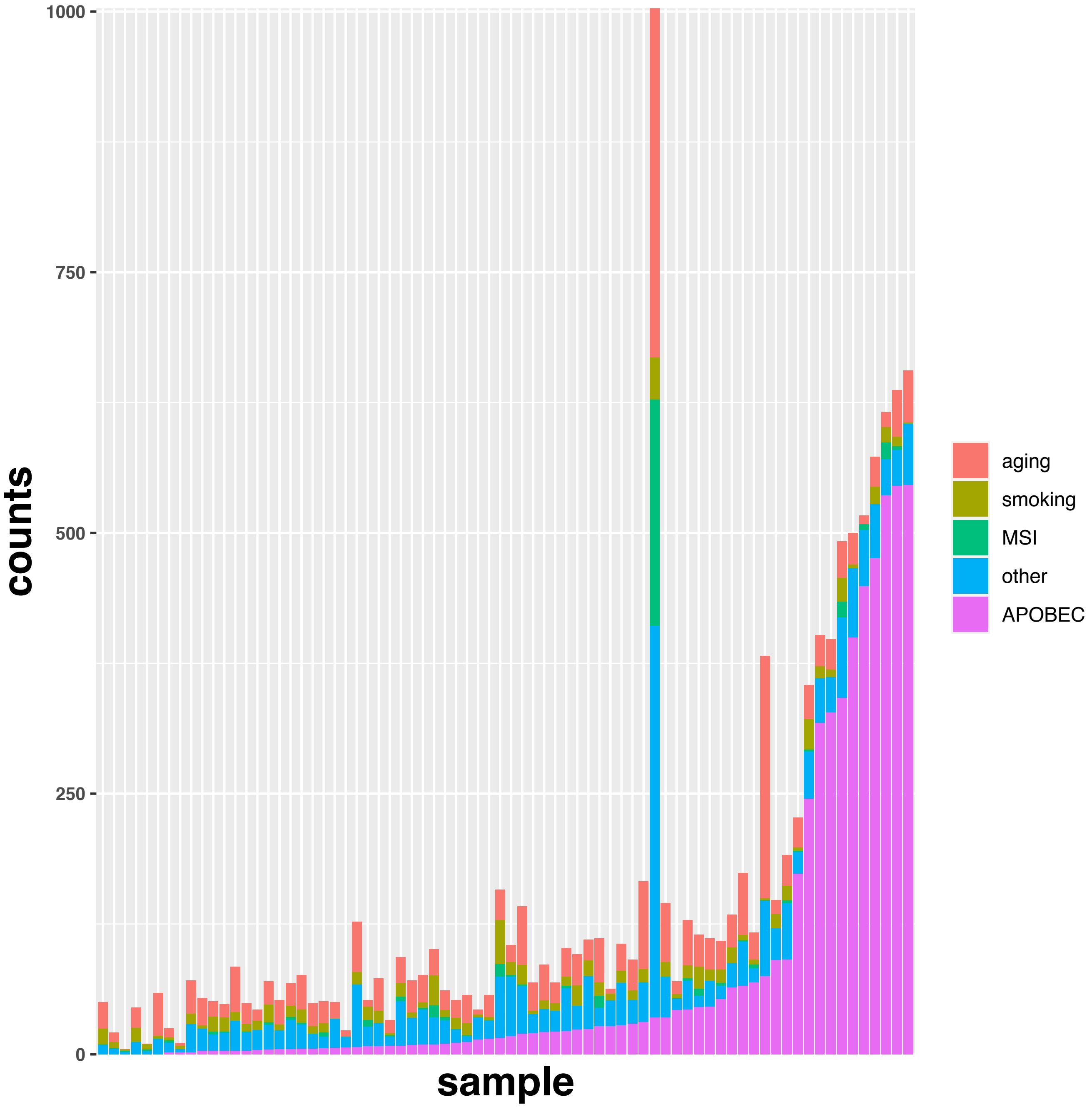

Figure S2. Expected (blue and red lines) vs actual (black dots) nonsynonymous/ synonymous ratio for APOBEC (red) and non-APOBEC (blue) mutations by viral gene.

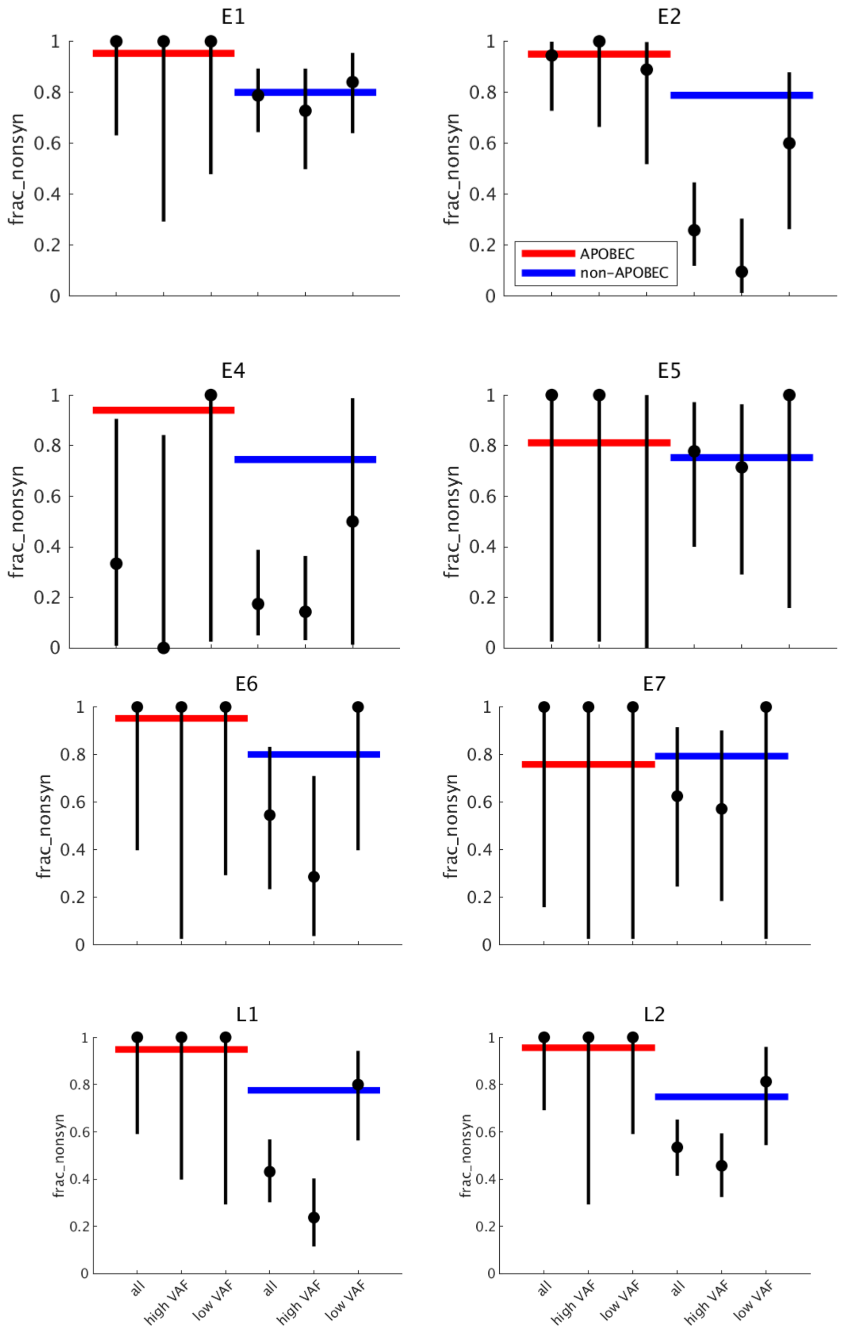

Supplement: Supplementary file 1 [file viruses-13-01666-s001.zip › supplementary material/Supplemental Figures copy.pdf]
